# Supplementary material for: Teosinte (Zea mays ssp parviglumis) growth and transcriptomic response to weed stress identifies similarities and differences between varieties and with modern maize varieties
Source: PLoS One. 2020 Aug 21;15(8):e0237715. doi: 10.1371/journal.pone.0237715 (PMC7444550; doi:10.1371/journal.pone.0237715)
Supplement: S1 Table — (DOCX) [file pone.0237715.s001.docx]

Supplemental File 1. Teosinte paper 12517

Per Sample Reads mapping data after RNASeq Analysis in CLC Bio Workbench.

| **Teosinte Line** | **Plot** | **Weedy/ Weed Free** | **Million Reads** | **%Reads Mapped in Pairs** | **% Reads Mapped in Broken Pairs** | **% Not Mapped** | **% Unique Fragments** | **Paired Distance** |
| --- | --- | --- | --- | --- | --- | --- | --- | --- |
| 789 | 208 | W | 33 | 63 | 16 | 20 | 80 | 136-261 |
| 789 | 105 | W | 35 | 63 | 17 | 22 | 78 | 127-258 |
| 789 | 207 | W | 22 | 64 | 15 | 21 | 79 | 126-254 |
| 789 | 106 | W | 35 | 63 | 15 | 21 | 79 | 125-253 |
| 789 | 405 | WF | 43 | 63 | 17 | 23 | 77 | 129-251 |
| 789 | 406 | WF | 29 | 63 | 16 | 21 | 79 | 133-255 |
| 789 | 301 | WF | 21 | 63 | 16 | 22 | 79 | 134-254 |
| 789 | 302 | WF | 37 | 63 | 16 | 21 | 80 | 136-257 |
| 812 | 401 | W | 30 | 62 | 17 | 20 | 80 | 136-258 |
| 812 | 402 | W | 37 | 65 | 17 | 21 | 79 | 130-253 |
| 812 | 111 | W | 20 | 63 | 16 | 21 | 79 | 126-262 |
| 812 | 112 | W | 23 | 62 | 16 | 21 | 79 | 92-269 |
| 812 | 308 | WF | 29 | 64 | 16 | 20 | 80 | 122-257 |
| 812 | 206 | WF | 29 | 61 | 16 | 20 | 80 | 136-260 |
| 812 | 307 | WF | 45 | 60 | 18 | 21 | 79 | 138-260 |
| 812 | 205 | WF | 23 | 63 | 16 | 21 | 79 | 120-256 |

Guided De Novo assembly statistics for 16 teosinte samples used in this study.

|  | **Count** | **Percent of Total Reads** | **Total Bases (Millions)** |
| --- | --- | --- | --- |
| **Reads** | 492,533,726 | -- | 49,153 |
| **Matched** | 393,970,826 | 80 | 39,305 |
| **No match** | 98,562,900 | 20 | 9,848 |
| **Contigs** | 140,292 | -- | 81 |
| **Reads in Pairs** | 316,722,810 | 64 | -- |
| **Broken Paired Reads** | 77,214,756 | 16 | -- |
