# Supplementary material for: Teosinte (Zea mays ssp parviglumis) growth and transcriptomic response to weed stress identifies similarities and differences between varieties and with modern maize varieties
Source: PLoS One. 2020 Aug 21;15(8):e0237715. doi: 10.1371/journal.pone.0237715 (PMC7444550; doi:10.1371/journal.pone.0237715)
Supplement: Attachment — Submitted filename: Review2020 plosone.docx [file pone.0237715.s005.docx]

Although the field experiments are old, the Authors showed morphological and transcriptomic responses of two teosinte lines (Ames 21812 and Ames 21789) in response to weed stress. The manuscript is well written. Experimental design and results come up with several conclusions. Most of them supported by data. The information could be exploited in the future to domesticate the elite varieties to deal with weed stress.

**Queries:**

Line 88. field conditions and to identify similarities and differences to (in) the transcriptomic response.

Query 1. How many times weeding was done to maintain the field weed-free, as weeds may come out within one week.

Query 2. As the weed species density varies in two years and biomass was similar, that hints that the weed species were different in two years, it would be better if authors can specify the weed species in each year, its number not the overall weed species, its density, and biomass, As each weed response can be different.

Query 3: Can Author justify a decrease in branch number in Ames 21812 in response to weed stress, from July (2.2) to 1.4 ( September).

Query 4. The authors did not mention the exact number of plants used for statistical analysis and phenotype parameters. If just 6 or 4 plants in 2013 and 2014 grown in a 3mX3m plot, it would be challenging to conclude the parameters.

Query 5. Have authors seen any difference in root length or diameter in response to weed stress, as root would be the most prone part to compete with weeds for nutrients?
