# Supplementary material for: Teosinte (Zea mays ssp parviglumis) growth and transcriptomic response to weed stress identifies similarities and differences between varieties and with modern maize varieties
Source: PLoS One. 2020 Aug 21;15(8):e0237715. doi: 10.1371/journal.pone.0237715 (PMC7444550; doi:10.1371/journal.pone.0237715)
Supplement: Attachment — Submitted filename: response II.docx [file pone.0237715.s007.docx]

Response to reviews II

Reviewer #1: General comment:

The manuscript entitled "Teosinte (Zea mays ssp parviglumis) growth and transcriptomic response to weed stress identifies similarities and differences between varieties and with modern maize varieties.." has been revised and re-submitted by Authors with the aim to evaluate the transcriptomic responses of teosinte (Zea mays ssp parviglumis), that is an ancestor of domesticated maize, to weed presence over two growing seasons.

Substantial modifications have been done by Authors to make the manuscript more conclusive and appealing as well as up to acceptable form.

Further, refining of the last sentence of Introduction as well as correction in Reference 10 may be done in galley proof.

Fixed the typos in both sentence and reference #10

Reviewer #2: The authors have addressed all the previous comments and integrated a few remarks in the revised version of the manuscript. But answers to some comments are just not queries, and those can be included in the discussion, which can address these arisen questions.

I am not sure what queries were not addressed. We did further refine our discussion as to the reason we focused on MtN21. It now reads:

Likewise, only the nodulin *MtN21* gene was up-regulated in both lines under weed stress. Thus, it might serve as a tool for identifying signaling networks involved in interference-sensing/response of teosinte, as it likely contains species-specific regulatory elements required for up-regulation in response to weeds. Other coordinately regulated gene sets could also serve to identify such elements but may be cultivar specific.
